# Mechanistic Insight into the Peptide Binding Modes to Two *M. tb* MazF Toxins

**DOI:** 10.3390/toxins13050319

**Published:** 2021-04-28

**Authors:** Ran Chen, Jie Zhou, Wei Xie

**Affiliations:** 1 MOE Key Laboratory of Gene Function and Regulation, State Key Laboratory for Biocontrol, School of Life Sciences, The Sun Yat-Sen University, Guangzhou 510006, China; chenran@mail.sysu.edu.cn (R.C.); zhouj288@mail2.sysu.edu.cn (J.Z.); 2 Key Laboratory of Tropical Marine Bio-Resources and Ecology, Guangdong Key Laboratory of Marine Materia Medica, Innovation Academy of South China Sea Ecology and Environmental Engineering, South China Sea Institute of Oceanology, Chinese Academy of Sciences, No.1119, Haibin Road, Nansha District, Guangzhou 511458, China

**Keywords:** tuberculosis, *Mycobacterium tuberculosis*, TA system, MazEF, antimicrobial peptide, cocrystal structure

## Abstract

Tuberculosis (TB) is a contagious disease caused by *Mycobacterium tuberculosis* (*M. tb*). It is regarded as a major health threat all over the world, mainly because of its high mortality and drug-resistant nature. Toxin-antitoxin (TA) systems are modules ubiquitously found in prokaryotic organisms, and the well-studied MazEF systems (MazE means “what is it?” in Hebrew) are implicated in the formation of “persister cells” in the *M. tb* pathogen. Here, we report cocrystal structures of *M. tb* MazF-mt1 and -mt9, two important MazF members responsible for specific mRNA and tRNA cleavages, respectively, in complexes with truncated forms of their cognate antitoxin peptides. These peptides bind to the toxins with comparable affinities to their full-length antitoxins, which would reduce the RNA-cleavage capacities of the toxins in vitro. After structural analysis of the binding modes, we systemically tested the influence of the substitutions of individual residues in the truncated MazE-mt9 peptide on its affinity. This study provides structural insight into the binding modes and the inhibition mechanisms between the MazE/F-mt TA pairs. More importantly, it contributes to the future design of peptide-based antimicrobial agents against TB and potentially relieves the drug-resistance problems by targeting novel *M. tb* proteins.

## 1. Introduction 

Tuberculosis (TB) is a contagious disease caused by *Mycobacterium tuberculosis* (*M. tb*). It is easily transmitted among people and about a quarter of the world’s population is estimated to be infected with this pathogen. In recent years, ten million people fall ill annually due to the TB infection around the globe [1]. More importantly, without proper treatments, the mortality rate from TB is quite high. In fact, TB is also the leading cause of death among transmitted diseases, even more lethal than HIV/AIDS. To make things worse, the drug-resistance problem of TB is a formidable challenge to TB care, as it is difficult to treat and takes longer to cure. Despite the global efforts to fight the disease, it continues to be a major public health threat. 

Toxin-antitoxin (TA) systems in prokaryotes are modules composed of a toxin and an antitoxin. They are expressed independently although transcribed from the same operon. Under normal growth conditions, they tend to form a stable complex, but their expression can be up-regulated to respond to stressful stimuli such as nutrient deficiency, antibiotic treatment, bacteriophage infection, etc. [2,3,4,5,6,7,8]. Eventually, dormant persister cells may form, characterized by slow cell growth or cell cycle arrests [9]. The TA system has been considered as one of the most promising antibacterial targets due to its wide occurrence and distribution in nearly all of the bacterial pathogens but not in eukaryotes [10,11]. The standard strain of *M. tuberculosis* H37Rv possesses more than 90 TA systems [12,13], including at least 30 functional TA operons. By contrast, *Mycobacterium smegmatis*, the non-pathogenic strain, harbors only three functional TA operons.

Over the past decades, a large number of divergent bacterial TA systems were identified and characterized. The type II TA families, whose components are both proteins, are the most studied type. Structural studies were carried out on the TA families including MazEF, CcdB, and YefM-YoeB, etc., which provided the interaction details of the protein pairs. All the MazF members are RNases, responsible for the cleavage of all types of RNAs participating in translation. Consequently, the activation of MazF RNases often leads to the inhibition of the translation process, reduction in metabolic rates, and eventually the formation of “persister cells” [10,14,15,16]. Because bacteria-caused infectious diseases are still the leading causes of human mortality worldwide, antimicrobial strategies targeting the toxin-antitoxin interactions are attractive, especially for the type II TA systems. However, inhibitors such as small molecules are too small to engage the large clefts between the two monomers of the MazF dimer. Consequently, peptides are more realistic due to their large sizes and versatile interactions with the toxins, and thus display higher affinities.

We previously determined the crystal structures of several members of the *M. tb* MazEF families and studied their detailed biochemical properties [17,18,19]. The structures of MazF-mt9 in its apo form and in complex with its antitoxin indicated that the antitoxin binds to a highly positively charged interface formed by the MazF-mt9 dimer, which we proposed to coincide with the binding site of tRNA substrates (PDBs 5WYG and 6A6X). Further mechanistic studies revealed that MazF-mt9 not only binds to its cognate antitoxin but also to the noncognate antitoxin MazE-mt1. We went on to determine the crystal structure of MazF-mt1 in various forms and characterized the association mode between the pair (PDBs 6L29, 6KYS, 6KYT, and 6L2A). Despite overall similarities between MazF-mt1 and MazF-mt9, their major structural differences reside in the dimer interface. While the MazF-mt9 interface is open with the relevant loops forming a disordered region, the MazF-mt1 interface is “locked” by swapped loops, which explains the extra effort needed for the antitoxin to bind and a relatively lower affinity for the latter. Our studies suggested that toxins and antitoxins from different families could crosstalk, and this mechanism may be used to counteract the toxicity of *M. tb* cells. 

In this study, we obtained the structures of MazF-mt9 and MazF-mt1 complexed with their corresponding antitoxin fragments, respectively. These peptides bind to the toxins with high affinities, accounting for the major driving forces of the TA pairings. We also conducted in-vitro experiments to show that the binding of the peptides would disable the toxins as RNases. The mechanistic and biochemical studies conducted in this study provide insight into the action modes of two important MazF family members and contribute to the future design of peptide-based antimicrobial agents against TB.

## 2. Results

### 2.1. The Heterologous Interactions between the MazEF Families 

**Figure 1:**
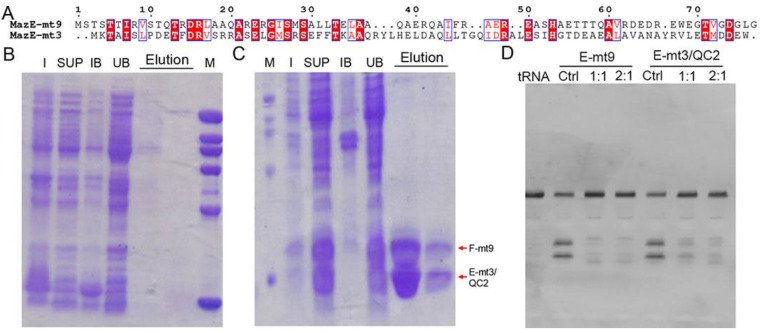
The investigation of the possible heterologous interactions between MazE-mt3 and MazF-mt9 and the engineering of MazE-mt3. (**A**) The sequence alignment between the MazE-mt9 sequence and that of MazE-mt3. (**B**,**C**) The first (**B**,**C**) second round of engineering of the MazE-mt3, and its subsequent co-expression profiles with MazF-mt9, which were named MazE-mt3/QC1 and MazE-mt3/QC2, respectively. M: molecular marker; I: Whole cell lysate of induced cells; SUP: the supernatant of ultrasonication after centrifugation; IB: the inclusion body after ultrasonication and centrifugation; UB: the unbound part from Ni-NTA column; Elution: the eluted fractions from the Ni-NTA column. (**D**) The inhibition against the tRNA-cleavage activity of the MazF-mt9 toxin by the engineered MazE-mt3/QC2 protein. Ctrl: MazF-mt9 without corresponding peptides. The ratios represented the molar ratios of toxin to various peptides used in the experiments.

We previously determined the cocrystal structures of the *M. tb* MazEF-mt1 and -mt9 complexes, and characterized their interactions by various techniques [18,19]. We showed that MazE-mt1 could be bound by MazF-mt9 with a moderate affinity both in vitro and in vivo but not vice versa. That is, MazE-mt9 does not bind MazF-mt1 although the sequences between the two antitoxins are very similar. When performing the sequence analysis on all the *M. tb* MazE sequences, we noticed that the N-terminus of MazE-mt3 shares homology with that of MazE-mt9, but differs in sequence at the C-terminus (Figure 1A). This discovery motivated us to test the idea of the possible non-orthogonal interactions between the two families. However, co-expression of MazE-mt3 with MazF-mt9 failed to produce either protein (data not shown), suggesting that the similarities between the N-termini were not the main factors mediating the interactions between the TA proteins. Consequently, we attempted to switch the last helix of MazE-mt3 (Val74-Trp82) to that of the α4-helix in MazE-mt9 (MazE-mt9/α4). We first changed the sequence to “WEGTVGDGLG” (named “MazE-mt3/QC1” in this study), a fragment of α4 with a length of 10 residues. However, the trial expression result was the same and no co-expressed proteins were evident on the SDS-PAGE gel (Figure 1B). We then changed the C-terminus of MazE-mt3 to the entire α4-helix with a sequence of “DEDREWEGTVGDGLG” (15 residues) (named “MazE-mt3/QC2” in this study). This complex (MazF-mt9/MazE-mt3/QC2) could be successfully co-expressed and purified to homogeneity, suggesting that the engineering did not affect the folding of the MazE-mt3 antitoxin and more importantly, conferred binding capacity to MazF-mt9, so the toxicity of the latter could be suppressed, and the host bacterium survived (Figure 1C). In accordance with this result, the tRNA-cleavage activity assays using *M. tb* tRNA^Lys(UUU)^ showed that the activity of the MazF-mt9 toxin could be effectively inhibited by the engineered MazE-mt3, the potency of which was comparable to that of the cognate antitoxin MazE-mt9 (Figure 1D, left). Therefore, the last helix alone is enough to mediate the binding event, whereas the rest of the sequence moderately contributes to the association of the TA complex, consistent with our previous observation [18].

### 2.2. The Interactions of MazE-mt9/α4 with MazF-mt9

**Figure 2:**
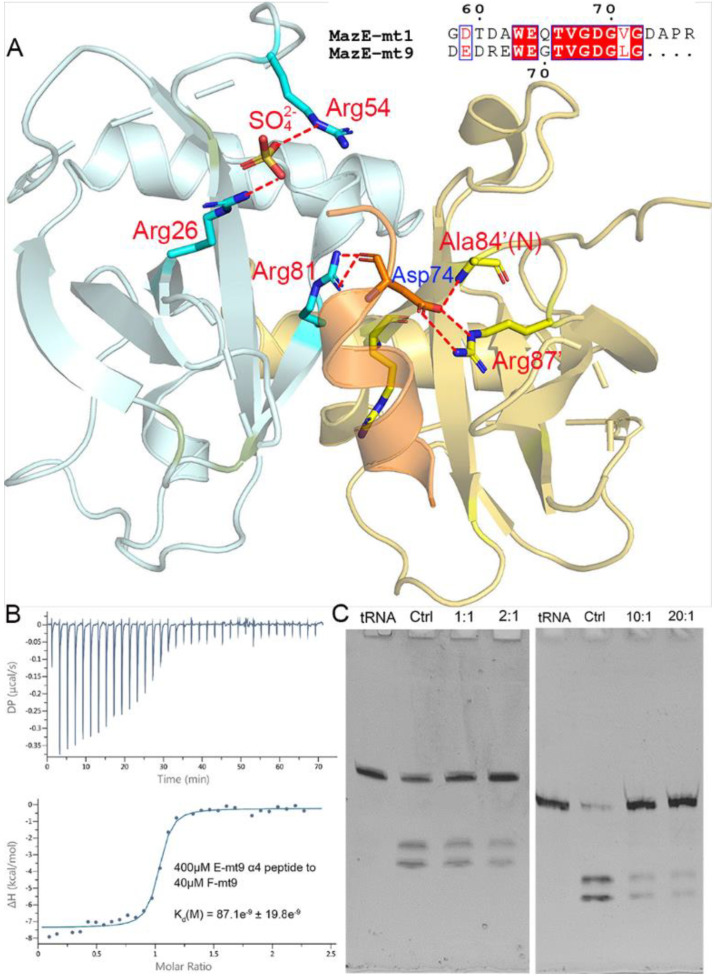
The interactions between the α4-helix of MazE-mt9 and the MazF-mt9 dimer. (**A**) The cocrystal structure (PDB 7DU4) showing the interactions of MazE-mt9 (α4) and the toxin. The hydrogen bonds and the salt bridges concerning the sulfate ion and Asp74 are indicated by the red dashed lines. The sequence alignment between MazE-mt9 (α4) and the C-terminus of MazE-mt1 was shown in the inset. (**B**) The quantitation of the MazE-mt9/α4 (15 residues) interactions as characterized by ITC. (**C**) The inhibitory effects of the α4 peptide against the tRNase activity of MazF-mt9.

The interactions with the MazF-mt9 toxin are mostly concentrated in the last helix of MazE-mt9 (α4), with the residue Asp74 conferring a dominant impact to the mutual binding (Figure 2A). The single mutation D74A would reduce the affinity by three-fold [18]. We synthesized the α4-peptide (Asp63–Gly77) and measured its affinity to the toxin by ITC. As expected, α4 bound to MazF-mt9 with a high affinity, and the k_d_ value was 87 nM, comparable to that of the full-length MazE-mt9 (k_d_ = 25 nM) [18] (Figure 2B). Next, the binding strength of the peptide to the toxin was also evaluated by its effects against the MazF-mt9 activity. At a molar ratio of 10:1, the α4-helix completely abolished the cleavage activity of the MazF-mt9 enzyme toward tRNA^Lys(UUU)^ (Figure 2C). 

**Figure 3:**
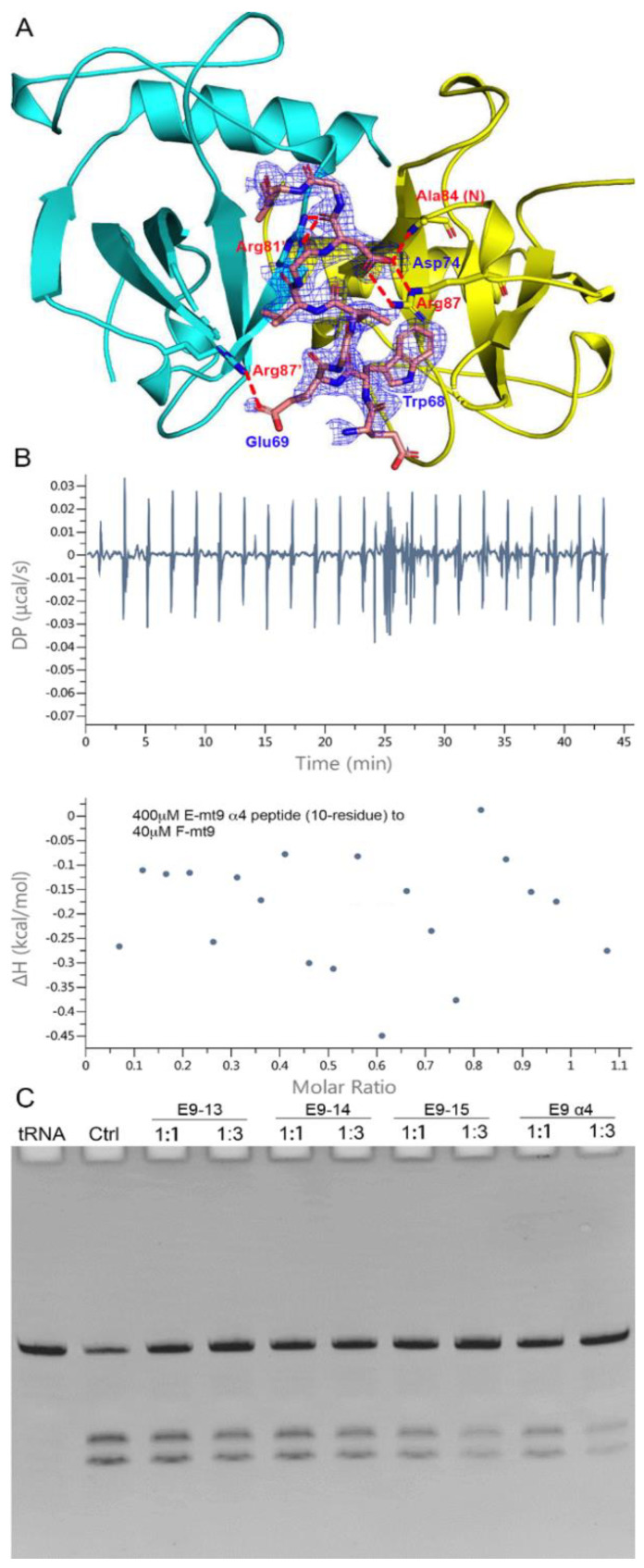
The recognition mechanism of the MazE-mt9 (α4) peptide by MazF-mt9. (**A**) The cocrystal structure of the MazF-mt9/α4 complex (PDB 7DU4), with the detailed interactions of Asp74 highlighted. The electron density of the OMIT map is shown by the blue mesh (contoured at 2σ). (**B**,**C**) The test and optimization of the peptide based on the α4-peptide, which was measured by the inhibition against the tRNase activity of MazF-mt9.

To reveal the interaction details between the pair, we obtained the structure of MazF-mt9 bound with the 15-residue α4-peptide (Figure 3A). The 2.2-Å high-resolution cocrystal structure (Table 1) shows that the peptide is bound in a mode similar to that of the last helix of the full-length MazEF-mt9 complex (PDB 6A6X), explaining the tight affinity of α4 to the toxin. The peptide binds in the interfacial groove of the MazF-mt9 dimer and forms a heterotrimeric structure, but the first four residues and the last residue were disordered. The rest of the structure assumes an overall helical conformation, and it retains most interactions observed in the full-length protein. In addition, the bulky Trp68 allows the helix to insert into the active site and makes hydrophobic contacts. An obvious difference between the two structures is the presence of a sulfate ion in the full-length structure, which came from the crystallization buffer. This sulfate is located at the contact point where the three molecules meet: Arg26 from chain A (MazF-mt9), Arg54’ from chain B (MazF-mt9’, the other monomer), and Leu76 from chain C (the antitoxin peptide) (Figure 2A). The anionic sulfate acts like a “molecular glue” to “pull” the macromolecules together and explains why the cocrystals of the MazEF-mt9 complex only grew in mother liquors containing sulfate. Interestingly, the peptide appeared to bind to the interface symmetrically with the isosteric residues Thr71Val72 acting as the central residues, which are located exactly in the middle of the resolved peptide (we only observed ten residues, Table 2). In the structure, the two central residues do not make any contacts with the toxin, and they are neighbored by glycines in sequence. The glycines, Gly70 and 73, are in turn neighbored by Glu69 and Asp74, respectively, which carry similar charges in nature. Additionally, the first and last visible residues are Glu67 and Leu76, respectively, similar in sizes as well. In short, the sequence arrangement pattern of the peptide is symmetrical, which prompted us to the idea that the peptide could be reversed without losing the affinity to MazF-mt9. 

The sequence and the binding mode of the peptide are interesting, and we therefore investigated the details of the complexes that it forms. We synthesized a series of peptides based on the original sequence (α4) and tested their influence on the activity of the toxin (Table 3). We found that the removal of the first four residues and the last residue (E9-α4 (10-residue)) from the peptide made a remarkable difference, as evidenced by its inability to inhibit the toxin, although these residues were disordered in the complex structure. The ITC titration also indicated that the 10-residue peptide lost its ability to associate with the toxin (Figure 3B). Additionally, the replacement of the central Thr71 residue by an aspartate (E9-15AA2) in the original 15-residue α4-peptide completely abolished the binding ability of the peptide. On the other hand, the replacement of the Val72 or Glu69 residues by an aspartate (E9-15AA3 and E9-15AA4, respectively) would barely affect the associations of the TA pairs. Two-residue substitutions normally led to total losses of the affinity (data not shown) except for the E9-13 and E9-14 peptides, both of which were based on the predecessor E9-15AA4 and MazF-mt9 showed reduced cleavage efficiencies when the peptides were at great excesses. E9-14 replaced Gly75 with a tryptophan on top of E9-15AA4, which created an even more symmetrical pattern with the WDGTVGDW residues in the sequence (Figure 3C). However, the addition of such a hydrophobic residue to the sequence also made the peptide more difficult to dissolve. Meanwhile, to fully assess the role of the bulky Trp68 residue in the formation and stability of the complex, which apparently makes no specific contacts with MazF-mt9 in the structure, we replaced it with two smaller residues (alanine) on top of E9-15AA4. The resulting peptide (E9-9) also severely impaired the binding affinity. These results may be attributed to the fact that Trp68 makes important hydrophobic interactions, namely with Val47, Val12’, and Val14’ of MazF-mt9. Furthermore, E9-16 completely reversed the sequence of α4, which did not show any association either. Lastly, we extended the original 15-residue sequence to 18 (E9-15), by further taking account the symmetry of the sequence into consideration (the two half sites are mirroring each other with the central TV residues). E9-15 demonstrated full activity, which took full advantage of the sequence symmetry (Figure 3C). Nevertheless, to completely understand the differential performance of the peptides, factors such as lengths, stabilities, as well as polarities should be taken into consideration. 

### 2.3. The Interactions of the MazE-mt1/α3 Helix with MazF-mt1 

In terms of the MazEF-mt1 TA system, our previous studies demonstrated that while η1 (Asp59-Arg76) did not bind to the cognate toxin, α3 (Thr42-Gly58) bound with a stronger affinity even greater than that of the full-length antitoxin. η1 was a small helix that follows α3, and the binding of η1 necessitates the conformational opening of the swapped loops (β1–β2) covering the MazF-mt1 dimer interface. Both the η1- and α3-helices make numerous interactions with MazF-mt1 in the cocrystal structure of the MazF-mt1/MazE-mt1 complex (PDB 6KYT), but the hindrance between the swapped loops and η1 requires extra energy to unravel these interlocked loops, which reduces the overall binding energy of the full-length antitoxin [19]. 

**Figure 4:**
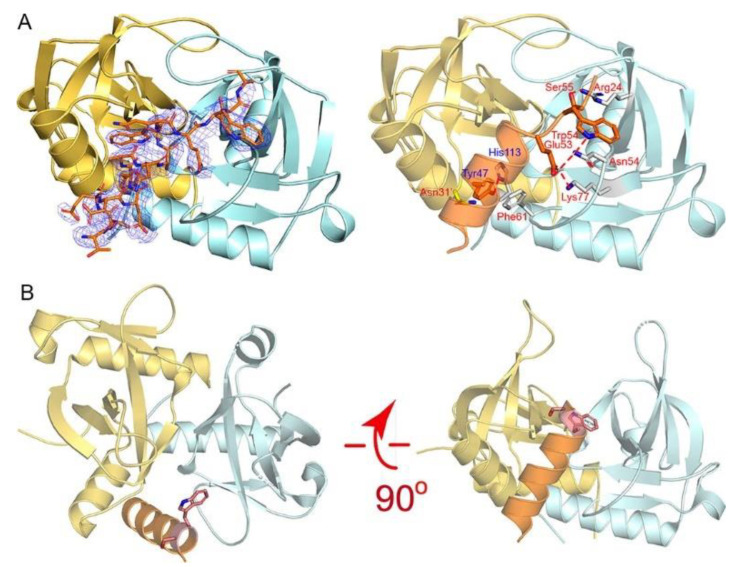
The recognition mechanism of the MazE-mt1 (α3) peptide by MazF-mt1. (**A**) The cocrystal structure of the MazE-mt1 (α3)/MazF-mt1 complex (PDB 7DU5) and detailed interactions are shown on the right. The electron density of the OMIT map is shown by the blue mesh and was contoured at 2σ. (**B**) The structure of the MazEF-mt1 complex with only the α3-peptide of the antitoxin being shown. Two orthogonal views of the latter are shown, and the Trp54Ser55 dipeptide is shown in sticks. Note that the view on the right was the same as that of Figure 4A.

To explore the possible binding mode between the α3-helix and MazF-mt1, we obtained the cocrystal structure of the MazF-mt1/MazE-mt1 (α3) complex (PDB 7DU5). Except for the two residues Trp54Ser55, the overall conformation and interactions are maintained in the cocrystal structure, when compared to that of the full-length MazE-mt1 complex (Figure 4A). The close-up of the interaction details revealed that the peptide complex is maintained by the interactions on residues Tyr47 and Glu53-Ser55 of the α3-peptide. However, the last three residues of the peptide failed to maintain the helical shape with Trp54 of the antitoxin swinging to the other side (Figure 4B). Additionally, the knot structure of MazF-mt1 became open due to the binding of the antitoxin peptide. Due to the extra energy needed to pry open this interface, the affinity of the MazF-mt1/MazE-mt1 complex is lower than the MazF-mt1/MazE-mt1 (α3).

On the determination of the structure of the MazF-mt1/α3 complex, we compared it to that of the MazF-mt1/RNA complex (PDB 5HJZ). Interestingly, we found that the 4-nt RNA ACCU occupies the site that α3 binds. Although the active site of MazF-mt1 is currently unclear, because the study concerning this PDB has not been published, we believe that this site at the dimer interface is very likely to be the RNase activity site for MazF-mt1. The tetranucleotide was probably from the cleaved substrate (the recognition site of MazF-mt1 is CU/ACC with “/” as the cleavage site), and the structure mimics the product-bound stage after the reaction. We therefore deduced that MazF-mt1 would be inhibited by the α3-helix as well. Similar to MazF-mt9, as we described above, we carried out the mRNA cleavage assay. We employed two substrates with different sequences and lengths by following the studies of Zhu et al., who reported that an unmodified, 15-nt RNA oligo containing the UAC motif could serve as the substrate for MazF-mt1 [20]. However, we failed to reproduce the mRNase activity of MazF-mt1 somehow, as the enzyme was incapable of generating the product even at excessive concentrations or prolonged incubation periods. Taken together, we found two peptides that could bind to MazF-mt1 and -mt9, respectively, with high affinities to their putative RNA-binding sites, and thus would inhibit the activities of their own cognate toxins.

## 3. Discussion

Tuberculosis has been a great threat since ancient history. In recent years, the TB crisis widely spreads out and has become more and more severe, especially in developing countries. In 2020, tuberculosis has become the number-one killer of all infectious diseases, which greatly adds to the financial burden of every country and threatens the health of humankind. The major reason for TB prevalence is the appearance of the drug-resistant *M. tb* strains, which render the first-line anti-TB drugs ineffective or even useless against their original targets. Therefore, treatments of this disease call for novel strategies against new targets. *M. tb* has more than 90 TA systems and 11 MazEF families, one of the salient features that distinguishes itself from other bacteria. Consequently, the idea of targeting MazEF TA systems was increasingly popular, and peptides against the RNase activities were designed and tested. 

In this study, we started by sequence analysis of the similarities between various *M. tb* MazE members, hoping to identify possible novel heterologous TA interactions. Due to its similarities at the N-terminus to that of MazE-mt9, we wonder if MazE-mt3 could interact with MazF-mt9. While unmodified MazE-mt3 protein was unable to bind and co-express with the MazF-mt9 toxin, we found that the last helix of MazE-mt9 was mainly responsible for the protein-protein interactions. On the other hand, the N-termini of MazE members are mainly involved in the formation of their dimers, which would allow them to bind the operators of their operons.

Next, we studied two fragments/peptides from the antitoxin of MazE-mt1 and -mt9, respectively, by obtaining the co-crystal structures with their cognate toxins. The structures revealed that the peptides adopt conformations similar to their full-length antitoxin proteins, i.e., they occupy the same putative substrate-binding sites. Additionally, the factor providing the binding affinity mainly resides in a single helix of the antitoxin while other elements are mainly structural. Therefore, these peptides bind so efficiently to their cognate toxins that they play a major role in mediating the protein-protein interactions.

To find out the contribution of each residue of the peptide to the binding affinity, we systematically studied the single, double, or even multiple substitutions of the residues in α4. We first found that the sizes of the peptides matter because the removal of disordered residues abolished binding. Next, we found the sequence requirement is quite stringent in that the replacement of seemingly non-essential residues also greatly affected the performance of the peptides. Only isosteric residues or residues of similar properties were effective (E9-13 and E9-14). Two- or three-residue substitutions were generally not allowed (E9-15AA5, E9-9, E9-10, and E9-12). Lastly, noticing that the dimeric enzyme binds the peptide in an almost symmetrical fashion, we tried out a series of peptides with “symmetrical” sequences in order to increase the affinity of the peptides further. To overcome the chiral problem of amino acids, we even synthesized several peptides with mixed L- and D-type amino acids to accomplish the exact structural symmetry (data not shown). These trials led to the discovery of a few candidates potentially useful for further testing (peptides E9-13, 14, and 15AA4), whose efficacies were demonstrated in the tRNA-cleavage activity assays. Taken together, our cocrystal structures and mutational studies provided in-depth details of the recognition mechanism by the toxins. Especially in the MazF-mt9 case, we obtained candidate peptides with different affinities, polarities, and sizes, which would aid in future peptide-based drug design against this toxin.

Although the sequences of the last helices of the two antitoxins (η1 and α4 in MazE-mt1 and -mt9, respectively) and the structures of the two toxins are quite similar, η1 in MazE-mt1 does not provide most of the binding energy for the MazF-mt1, unlike α4. This came as a surprise, initially. The structural basis for this phenomenon is that MazF-mt1 forms swapped loops across the interface. These loops need to open up for the antitoxin to enter. In contrast, MazF-mt9 harbors intrinsic disorder at the corresponding regions, and it would be relatively easy for its cognate antitoxin to bind. Thus, the major binding energy to MazF-mt1 mainly comes from α3 of MazE-mt1, and this result becomes more evident after the superimposition of the MazF-mt1/MazE-mt1 complex with that of MazF-mt1 cocrystallized with RNA (PDB 5HJZ). The α3-helix poses severe clashes against the 4-nt RNA fragment, which is a putative substrate/product. Therefore, the RNA-binding site, as shown in PDB 5HJZ, very likely overlaps with that of α3 of MazE-mt1. Although we failed to prove this point directly in our subsequent RNA-cleavage experiment due to unknown reasons, the strong affinity of α3 toward MazF-mt1 and the structural coincidence with the RNA fragment both suggested that the α3-helix would prevent the binding of the potential RNA substrate as well. Therefore, MazF proteins are intriguing molecules in evolving the substrate-binding sites and recognition modes despite their small sizes. Although similar in overall sequences and structures, MazFs appear to harbor different potential RNA-binding sites, which contribute to their wide substrate specificities. 

We lastly tested the antimicrobial efficacies of the peptides by inhibiting the growth of the H37Ra strain. While the peptides were efficient in inhibiting the activities of the toxins in vitro, our preliminary results showed that their in-vivo activities were poor (data not shown). The idea of using antitoxin fragments as potential bactericidal agents was first tested on the *B. anthracis* PemIK TA system, where the structural and biochemical data of its binding mode was employed to inhibit the TA interactions. PemK cleaves single-stranded RNAs, but this activity is neutralized by PemI. A peptide mimicking the C-terminal region of the antitoxin was designed by Chopra and coworkers, which decreased the ribonuclease activity of the toxin [21]. Recently, Kang et al. designed peptides that triggered *Streptococcus pneumoniae* cell death based on their structural studies and the recognition mechanism of the HigBA TA system [22]. Using a stapling strategy, they also achieved the penetration of the peptides across the membranes with an MIC_50_ smaller than 6.25 μM. These peptides targeting the direct active or allosteric sites of the toxins accomplished a series of successes in inhibiting or killing the bacterial pathogens. In retrospect, one of the reasons for the failure of our in-vivo experiments could be the limited capability of the peptides to penetrate the membrane due to their relatively large sizes, which would be a major obstacle for their in-vivo applications. Additionally, stabilities of the peptides could also be an issue, and whose pharmacokinetic data are currently unavailable. Chemical modifications such as cyclization or PEGylation of the peptides are currently underway to increase their half-lives as well as chances to reach the cytoplasm and to boost their efficacies.

## 4. Materials and Methods

### 4.1. Cloning, Expression, and Purification of the Proteins

The preparation of MazF-mt1 and MazF-mt9 proteins were described in previous papers [17,19]. The gene encoding MazE-mt3 was inserted into cloning site 1 (MCS1) of the modified pETDuet1 vector (Novagen, Madison, WI, USA) to give pETDuet1/mazE-3. The protein was expressed similarly to that of the MazF-mt1 and MazF-mt9 proteins and isolated by a two-step purification protocol using affinity and anion-exchange purification techniques. The N-terminal 6 × His-tag was cleaved off, and the target protein was obtained by passing through the Ni-NTA column a second time and collecting the unbound fractions. The co-expression vector with mazF-mt9 was achieved by inserting mazF-mt9 into MCS2 of the modified pETDuet1 to produce pETDuet1/mazE-mt3/mazF-mt9. Mutants of these genes were generated by the QuikChange method (Agilent, Santa Clara, CA, USA) using this vector as the template.

### 4.2. Crystallization, Data Collection, and Structure Determination

The initial screens for MazF/peptides cocrystals were manually set up using the sitting-drop vapor-diffusion method. The sample was mixed with the well solution at a 1:1 ratio (*v*/*v*). The crystals were acquired under similar conditions to those of the WT apo-proteins. Basically, the MazF-mt1 protein was concentrated to 2.0 mg mL^−1^, and mixed with the α3-peptide at a molar ratio of 1:5. The cocrystals of the MazF-mt1 complex were obtained at 20% PEG 3350, 0.1 M NaOAc pH 5.0, and 0.1 M NaCl. The MazF-mt9 protein was concentrated to 3.0 mg mL^−1^ and mixed with the α4-peptide at a molar ratio of 1:10. The cocrystals of the MazF-mt9 complex were obtained at 1.5 M NaCl, 0.2 M (NH_4_)_2_SO4, and 0.1 M NaOAc pH 5.0. The cocrystals typically needed two days to appear and 3–5 days to grow to full sizes. All the fully grown crystals were soaked in a freshly made cryoprotective solution containing all the components of the reservoir solution plus 20% (*v*/*v*) glycerol. The soaked crystals were mounted on nylon loops and flash-cooled in liquid nitrogen. Diffraction datasets were collected at beamline 19U1 (BL19U1) at the Shanghai Synchrotron Radiation Facility (SSRF, Shanghai, China) and were processed with the program *HKL3000* [23].

The structures were solved by molecular replacement using the Phaser program with the coordinates of PDB 6KYS and 5WYG as the search models. On the basis of the solution, the models were further rebuilt manually with COOT according to the electron density map [24]. Multiple cycles of refinement alternating with model rebuilding were carried out with PHENIX.refine [25]. The final model was validated by molprobity [26]. The structural figures were produced with PyMOL (www.pymol.org, accessed on 1 March 2021). All data collection and refinement statistics are presented in Table 1.

### 4.3. tRNA Cleavage Assays

The preparation of the *M. tb* tRNA^Lys(UUU)^ was described in a previous protocol [17]. When carrying out the MazF-mt9 cleavage assays in the presence of the MazE-mt9 peptides or mutants, 25 pmol of peptides were mixed with 50 pmol MazF-mt9 or indicated amounts of the toxin. All mixtures were incubated on ice for 30 min to allow the formation of the complex. 0.6 μL of the complex was added to a reaction mixture of 20 mM HEPES (pH 7.5), 50 mM potassium chloride, 1 mM DTT, and 8 pmol of *M. tb* tRNA^Lys(UUU)^ substrate, and incubated at 37 °C. The reactions were stopped after 30 min by adding the 2 × formamide gel-loading buffer (95% *w*/*v* formamide and 50 mM EDTA). The samples were denatured at 95 °C for 5 min before electrophoresis in a 15% Urea-PAGE gel containing 7 M urea, followed by ethidium-bromide staining. 

### 4.4. Isothermal Titration Calorimetry (ITC)

ITC experiments were conducted at 25 °C using a PEAQ ITC titration calorimeter (Malvern Instruments, Malvern, UK). To exactly match the buffer compositions, the MazE and MazF proteins or peptides were dialyzed against the same buffer containing 20 mM Tris-HCl (pH 8.0), 150 mM NaCl, and 1 mM DTT. The MazE-mt9 α4 (Asp63-Gly77: DEDREWEGTVGDGLG), MazE-mt1 α3 (Thr42-Gly58: TLEDDYANAWQE WSAAG), and other peptides were synthesized by DGpeptides Co., Ltd.(Hangzhou, China) (Table 3). The concentrations of the peptides or protein were adjusted according to the binding profiles. The first injection of 0.4 μL was followed by 18–35 injections of 1 μL drops. The MICROCAL ORIGIN software was used to determine the number of binding sites and the model that produced good fits.

## Figures and Tables

**Table 1:** Data collection and refinement statistics.

PDB IDs	MazF-mt9-α4	MazF-mt1-α3
	7DU4	7DU5
Data collection	SSRF BL19U1	SSRF BL19U1
Wavelength	0.979	0.979
Space group	P6_5_	P6_5_22
Cell dimensions		
a, b, c (Å)	74.61, 74.61, 104.35	83.27, 83.27, 141.27
α, β, γ (°)	90.0, 90.0, 120.0	90.0, 90.0, 90.0
Resolution (Å)	50.0–2.18 (2.26–2.18) ^a^	41.64–2.65 (2.78–2.65)
R_merge_^b^ (%)	20.5 (75.9)	6.1 (55)
I/σ_(I)_	13.2 (4.2)	30.5 (6.5)
Completeness (%)	100 (99.9)	99.8 (99.6)
Redundancy	19.2 (16.2)	18.8 (20.1)
Refinement		
Resolution (Å)	37.31–2.18 (2.32–2.18)	41.64-2.65 (3.03–2.65)
No. reflections	16599	8909
R_work_^c^/R_free_^d^	0.183/ 0.225	0.254/0.280
No. atoms		
Protein	1558	1746
Ligand (peptide)	74	127
Water	117	0
B-factors (Å^2^)		
Protein	31.4	81.3
Ligand (peptide)	45.8	90.1
Water	38.7	-
R.m.s deviations		
Bond lengths (Å)	0.007	0.006
Bond angles (°)	0.98	1.10
Ramachandran favored (%)	99.06	95.90
Allowed (%)	0.94	4.10
Outliers (%)	0	0

^a^: Values in parentheses are for the highest-resolution shell. ^b^: R_merge_ =Σ |(I − <I> )|/σ(I), where I is the observed intensity. ^c^: R_work_ = Σ_hkl_ ||Fo| − |Fc||/ Σ_hkl_ |Fo|, calculated from working data set. ^d^: R_free_ is calculated from 5.0% of data randomly chosen and not included in refinement.

**Table 2:** The summary of the interactions between the peptides and their cognate MazF-mt toxins.

MazF-mt9-α4			MazF-mt1-α3		
Peptide	MazF-mt9 (Chain A)	MazF-mt9 (Chain B)	Peptide	MazF-mt1 (Chain A)	MazF-mt1 (Chain B)
Gly70 (O)	Arg87 (NH_2_)		Tyr47 (stacking)	Phe61	
Glu69		Arg87’ (NH_2_)	Tyr47 (OH)	His113 (ND_1_)	Asn31’ (ND_2_)
Asp74 (OD_2_)	Ala84 (N)		Glu53 (OE_2_)	Asn54 (ND2)	
Asp74 (OD_1_, OD_2_)	Arg87 (NE, NH_2_)		Glu53 (OE_1_)	Lys77(NZ)	
Asp74 (O)		Arg81’(NH_1_, NH_2_)	Trp54 (NE_1_)	Asn54 (ND_2_)	
			Trp54 (stacking)	Asn54	
			Ser55 (OG)	Arg24 (NH_2_)	
			Ser55 (O)	Arg24 (NH_1_)	

**Table 3:** List of peptides used in this study.

Name	Sequence	Binding Affinity
E9 α4 (10 residue)	EWEGTVGDGL	No binding
E9 α4	DEDREWEGTVGDGLG	Strong binding
E1 α3	TLEDDYANAWQEWSAAG	Strong binding to MazF-mt1
E9-9	DEDREAADGTVGDGLG	No binding
E9-10	DEDRAAADGTVGDGLG	No binding
E9-12	DEDRAAADGTDGDGLG	No binding
E9-13	DEDRLWDGTVGDGLG	Strong binding
E9-14	DEDREWDGTVGDWLG	Strong binding
E9-15	DEDREWDGTVGDWERDED	Strong binding
E9-16	GLGDGVTGEWERDED	No binding
E9-15AA2	DEDREWEGDVGDGLG	No binding
E9-15AA3	DEDREWEGTDGDGLG	Strong binding
E9-15AA4	DEDREWDGTVGDGLG	Strong binding
E9-15AA5	DEDREGDGTVGDGLG	No binding
E9-15AA6	DEDRLGDGTVGDGLG	No binding
E9-15AA7	DEDRLGDGTTGDGLG	No binding

## Data Availability

All data relevant to this study are supplied in the manuscript are available from the corresponding author upon request. Coordinates and structure factors are deposited in the Protein Data Bank with the PDB entries: 7DU4 and 7DU5.
